# The transepicondylar axis or the posterior condylar axis: Which is the best reference for femoral component rotation in robotic-assisted total knee arthroplasty?

**DOI:** 10.1186/s42836-025-00362-7

**Published:** 2026-02-27

**Authors:** Qing-Da Wei, Hao-Ming An, Yun-Hao Tang, Ming-Feng Li, Rui Li, Wei Chai

**Affiliations:** 1 Senior Department of Orthopedics, the Fourth Medical Center, PLA General Hospital, Beijing, 100037 China; 2 Chinese PLA Medical School, Beijing, 100048 China; 3 School of Medicine, Nankai University, Tianjin, 300071 China; 4 National Clinical Research Center for Orthopedics, Sports Medicine and Rehabilitation, Beijing, 100048 China

**Keywords:** Robotic-assisted surgery, Total knee arthroplasty, Femoral component rotation, Landmark

## Abstract

**Background:**

For robotic-assisted total knee arthroplasty (TKA), accurate identification of anatomical landmarks directly affects the displayed value for femoral component rotation. This study aimed to quantify the inter-surgeon consistency of the TEA-reference angle (the angle between the transepicondylar axis and the femoral component axis) and the PCA-reference angle (the angle between the posterior condylar axis and the femoral component axis).

**Methods:**

The anatomical data of 56 patients who underwent robotic-assisted TKA at our institution were analyzed. Two surgeons independently identified the transepicondylar axis (TEA) and posterior condylar axis (PCA) landmarks on the 3D femoral models generated by the MAKO TKA system. The TEA-reference angle was recorded as α with the PCA-reference angle standardized to 0°, and the PCA-reference angle was recorded as β with the TEA-reference angle standardized to 0°. The measured values were α_1_β_1_ for Surgeon-1 and α_2_β_2_ for Surgeon-2. The differences between surgeons for α (∆α = α₁ – α₂) and β (∆β = β₁ – β₂) were calculated. The values of α and β are defined as positive for external rotation and negative for internal rotation.

**Results:**

The inter-surgeon intraclass correlation coefficient (ICC) for α was 0.761 (95% CI: 0.592–0.860), and that for β was 0.943 (95% CI: 0.902–0.966). The absolute difference between surgeons (∆α) was > 2° in 15/56 (26.8%) patients and ≤ 1° in 24/56 (42.9%) patients. With respect to ∆β, 3/56 (5.4%) patients had a difference > 2°, whereas 45/56 (80.4%) patients had a difference ≤ 1°.

**Conclusion:**

The inter-surgeon consistency of the PCA was significantly greater than that of the TEA in robotic-assisted TKA planning. To mitigate the risk of inappropriate femoral component rotation, surgeons should verify landmark positions, particularly in patients with anatomical abnormalities of the distal femur, and consider cross-referencing both axes.

Video Abstract

**Supplementary Information:**

The online version contains supplementary material available at 10.1186/s42836-025-00362-7.

## Introduction

Surgical robots are efficient tools that have changed the total knee arthroplasty (TKA) process, owing to their use in preoperative planning and during surgery [1–3]. However, an issue that is often overlooked is that the osteotomy plan displayed by the robot depends on the landmarks identified by the engineer (product specialist). The different landmark identification may affect the surgeons’ judgment of the osteotomy parameters. Robotic precision for coronal alignment restoration has been confirmed in several studies [4, 5]. However, few studies [6] have focused on femoral component rotation in robotic-assisted TKA, which may affect knee biomechanics [7].

In robotic-assisted TKA, the surgical transepicondylar axis (TEA, a line connecting the medial epicondylar sulcus with the most prominent point of the lateral epicondylar sulcus [8]) and the posterior condylar axis (PCA, a line connecting the most posterior points of the medial and lateral femoral condyles of the femur [9]) are commonly used to determine the femoral component rotation. Which parameter is more suitable as a reference line in robotic-assisted TKA remains to be investigated.

Some studies have reported that the TEA is more difficult to identify [10–12]. Some researchers believe that the PCA is more consistently identifiable among different surgeons [13, 14]. Our study of robotic-assisted TKA osteotomy parameters also confirmed that the coefficient of variation for the TEA is greater than that of the TEA [15], which suggests that the identification of the landmark location has a direct effect on the osteotomy parameters. However, the magnitude of its impact on the judgment of femoral component rotation remains unknown.

Therefore, this study aimed to determine the effects of landmark identification by different surgeons on the consistency of the TEA-reference angle (the angle between the TEA and the femoral component axis) and PCA-reference angle (the angle between the PCA and the femoral component axis) within the MAKO TKA system.

## Methods

In accordance with the Declaration of Helsinki, the scientific and ethical committees of our institution approved this study (2024KY074-KS001). We reviewed the distal femoral model data stored in the MAKO TKA system (Stryker, USA) at our institute. All included patients underwent TKA with the MAKO system between June 2021 and March 2024. The exclusion criteria for this study were (1) severe flexion contracture deformities (> 15°); (2) severe varus or valgus deformities (> 15°); and (3) patients whose modeling data were missing from the MAKO TKA system. Ultimately, 56 patients with unilateral primary knee osteoarthritis were included in the study. Informed written consent was obtained from all participants.

### Landmark identification

**Fig. 1:**
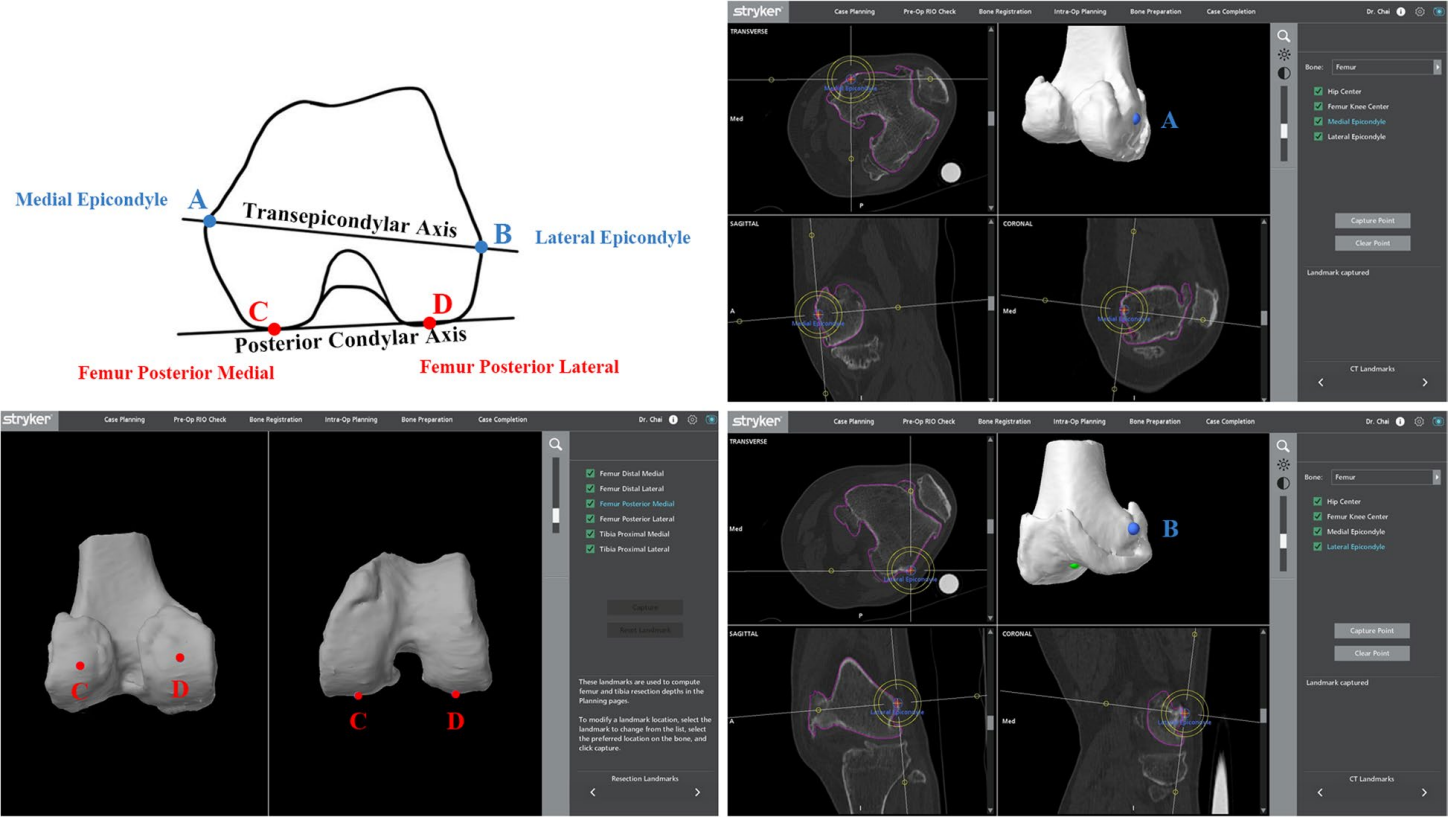
The standard of landmark identification. In the 3D model of the distal femur reconstructed by the MAKO TKA system, the bony landmark points were marked. The surgical transepicondylar axis is the line between the sulcus of the medial epicondyle (blue point A) and the most prominent point of the lateral epicondyle (blue point B). The posterior condylar axis is the line connecting the lowest points of the medial (red point C) and lateral posterior (red point D) condyles of the femur. All landmarks were identified twice and then averaged

In the 3D model of the distal femur reconstructed by the MAKO TKA system (RIO 1.0), the bony landmark points were marked by two experienced surgeons following previously published methods [8, 9], after which each reference axis was determined. The surgical transepicondylar axis (TEA) [8] is the line between the sulcus of the medial epicondyle (point A in Fig. 1) and the most prominent point of the lateral epicondyle (point B in Fig. 1). The posterior condylar axis (PCA) [9] is the line connecting the most posterior points of the medial (point C in Fig. 1) and lateral femoral condyles (point D in Fig. 1) of the femur. All landmarks were identified twice and then averaged.

### Experimental procedure

**Fig. 2:**
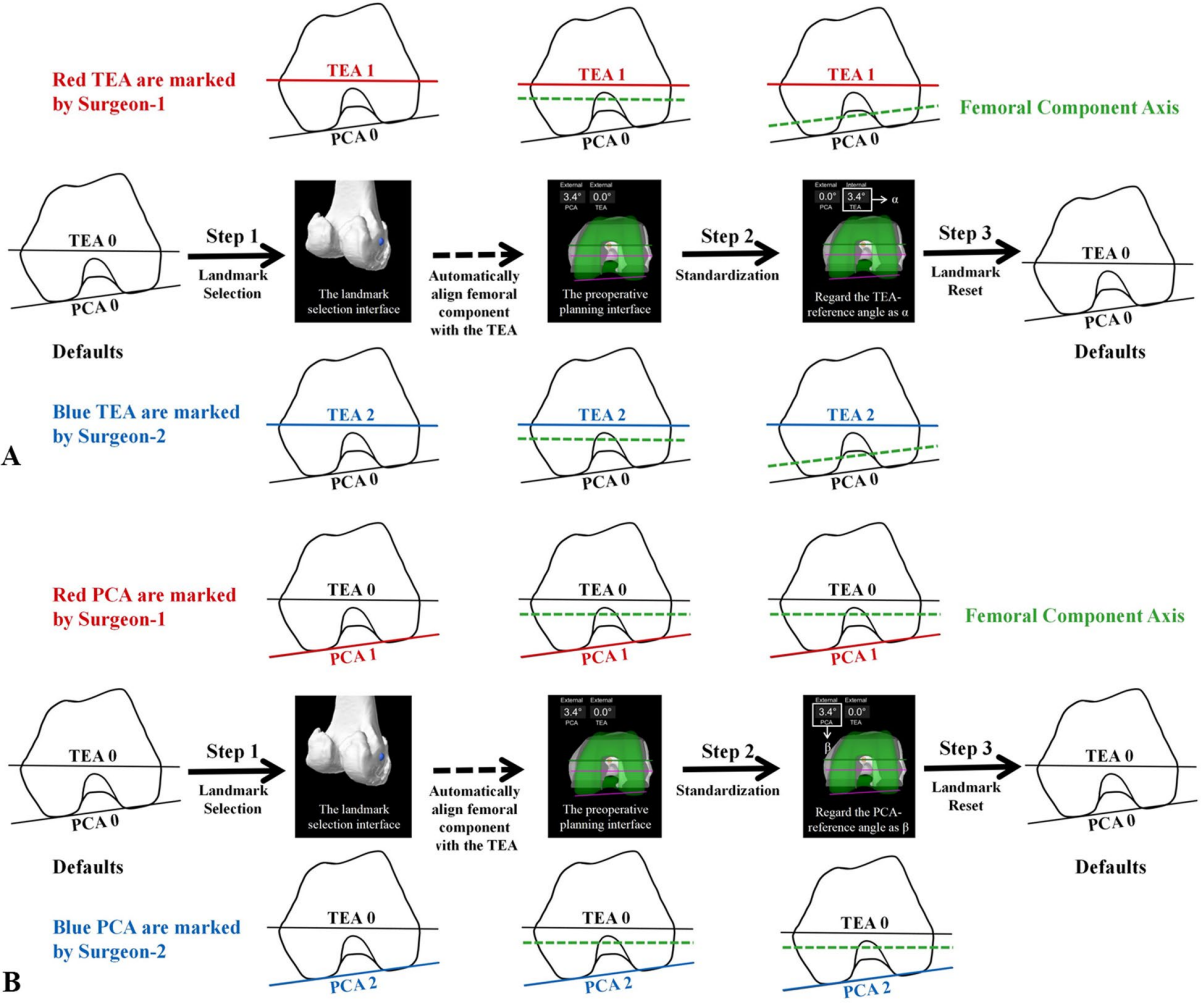
Workflow of landmark identification by different surgeons. In the landmark selection interface of the MAKO TKA system (Step 1), the landmarks of the TEA/PCA were obtained. Then, the MAKO TKA system automatically aligns the femoral component axis with the TEA. (Step 2) To obtain the TEA/PCA-reference angle (α/β), the femoral component axis was adjusted to be parallel to the PCA/TEA. (Step 3) After each operation, the selected points were changed to random landmarks. The above procedures were repeated by two surgeons

All measurements were performed postoperatively using the patient’s anatomical model within the MAKO TKA system without affecting the actual surgery. The femoral component axis is defined as the longitudinal axis parallel to the posterior condylar line of the Triathlon PS (Stryker) component by the MAKO TKA system [16]. The TEA-reference angle (the angle between the TEA and the femoral component axis) and PCA-reference angle (the angle between the PCA and the femoral component axis) values were displayed on the preoperative planning interface. The MAKO product specialist assisted with the system operation but did not participate in landmark identification for this study. The procedure was as follows (Fig. 2):

The TEA was selected first in the landmark selection interface. (Step 1) Surgeon-1 marked the medial and lateral epicondyles to obtain the respective TEA-reference and PCA-reference angles while keeping the default position of the PCA. The MAKO TKA system automatically adjusts the position of the femoral component after selecting the TEA/PCA (align the femoral component axis with the TEA [16]). This is why we performed the “standardization” operation uniformly: (Step 2) we adjusted the femoral component axis to be parallel to the PCA, and the TEA-reference angle was recorded as α when the PCA-reference angle = 0°. (Step 3) After each operation, the selected points were changed to random landmarks. The procedure was repeated for Surgeon-2. The measured values were α_1_ for Surgeon-1 and α_2_ for Surgeon-2.

The PCA was selected secondly in the landmark selection interface. (Step 1) Surgeon-1 marked the medial and lateral posterior femoral condyles to obtain the respective TEA-reference and PCA-reference angles while keeping the default position of the TEA. (Step 2) The data were standardized such that we adjusted the femoral component axis to be parallel to the TEA, and the PCA-reference angle was recorded as β when the TEA-reference angle = 0°. (Step 3) After each operation, the selected points were changed to random landmarks. Surgeon-2 repeated the procedure. The measured values were β_1_ for Surgeon-1 and β_2_ for Surgeon-2.

To minimize bias, Surgeon-1 and Surgeon-2 operated with mutual blinding. In addition, to circumvent the interference of the previous location, after each operation, the landmark selection interface was reset by a product specialist, and the selected points were changed to random landmarks. The differences between surgeons for the TEA-reference angle were calculated as ∆α = α₁ – α₂, and those for the PCA-reference angle were calculated as ∆β = β₁ – β₂. The values of α and β are defined as positive for external rotation and negative for internal rotation.

### Statistical analysis

Patient characteristics were subjected to statistical analysis, and a *P*-value < 0.05 indicated statistical significance. We performed *t*-tests to compare continuous variables and the chi-square test or Fisher’s exact probability test to compare categorical variables. The inter-surgeon reliability for α and β was assessed using a two-way random-effects model, absolute agreement, single-measure intraclass correlation coefficient (ICC(2,1)), and 95% confidence intervals (CIs). The standard error of measurement (SEM) and minimal detectable change (MDC) at the 95% confidence level (MDC = 1.96√2 SEM) were computed. The distributions of α and β values were tested for normality using the Kolmogorov–Smirnov test (*P* > 0.05). The proportions of cases with specific ∆α and ∆β magnitudes are reported as counts (n/N) and percentages. Bland–Altman plots were generated to visualize the limits of agreement between surgeons for α and β. Statistical analyses were performed using SPSS (v27.0), and the figures were drawn with GraphPad Prism, version 9.5 for Windows (GraphPad software).

## Results

The characteristics of the patients are shown in Table 1.

**Table 1:** Characteristics of the patients (*n* = 56)

Characteristic	Value
Age (yrs)	67.69 ± 5.27 (55–79)
BMI (kg/m^2^)	26.40 ± 2.54 (19.14–31.05)
Sex (*n*, %)	
Men	19 (33.9%)
Women	37 (66.1%)
Side (*n*, %)	
Left	26 (46.4%)
Right	30 (53.6%)
Preoperative HKA (°)	7.24 ± 5.19 (−14.8–14.9)
Preoperative LDFA (°)	88.16 ± 3.58 (74.8–100.0)
Preoperative MPTA (°)	84.75 ± 3.29 (75.2–95.9)

Quantitative data are presented as means ± SDs, with the range in brackets; categorical data are presented as numbers, with the corresponding percentages in brackets. BMI, body mass index; HKA, hip-knee angle, positive for varus, negative for valgus; LDFA, lateral distal femoral angle; MPTA, medial proximal tibial angle

**Fig. 3:**
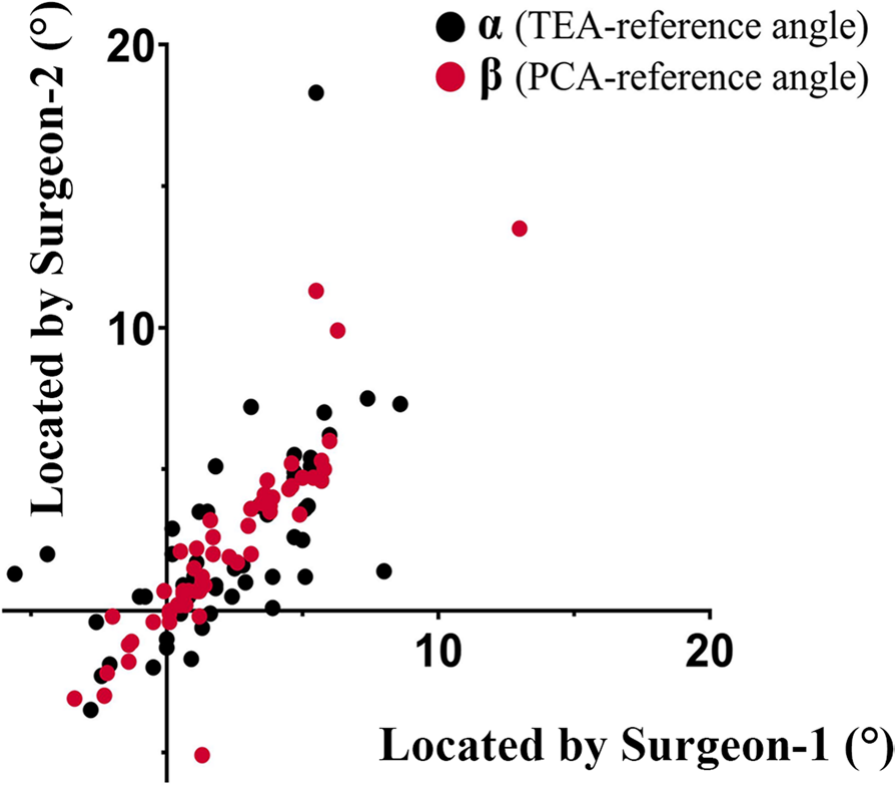
The values of α and β measured by the two surgeons. α values (black points) are the TEA-reference angle (angle between the transepicondylar axis and the femoral component axis) when the PCA-reference angle (angle between the posterior condylar axis and the femoral component axis) was adjusted to 0°. β values (red points) are the PCA-reference angle when the TEA-reference angle was adjusted to 0°. The values of α and β are defined as positive for external rotation and negative for internal rotation

**Fig. 4:**
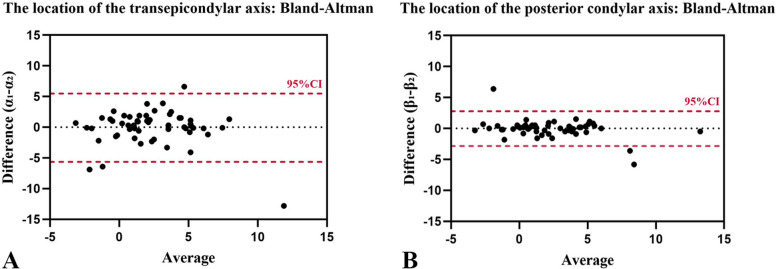
Bland–Altman plots for the inter-surgeon reliability of the femoral rotation angle. The α value is the TEA-reference angle (the angle between the transepicondylar axis and the femoral component axis) when the PCA-reference angle (the angle between the posterior condylar axis and the femoral component axis) is adjusted to 0°. The β value is the PCA-reference angle when the TEA-reference angle is adjusted to 0°. The measured values were α_1_β_1_ for Surgeon-1 and α_2_β_2_ for Surgeon-2. **(A)** Reliability for the TEA-reference angle. The X-axis shows the difference between surgeons (α_1_-α_2_), and the Y-axis shows the mean of the two measurements [(α_1_ + α_2_)/2]. **(B)** Reliability of the PCA-reference angle. The X-axis shows the difference between surgeons (β_1_-β_2_), and the Y-axis shows the mean of the two measurements [(β_1_ + β_2_)/2]. The red dashed lines represent the 95% limits of reliability

The values of α and β measured by the two surgeons are detailed in Fig. 3. The data above conformed to a normal distribution, as indicated by the results of the Kolmogorov–Smirnov test (*P* > 0.05). The Bland–Altman plots (Fig. 4) revealed wider limits of agreement for α (−4.9° to 4.7°) than for β (−2.2° to 2.1°).

**Fig. 5:**
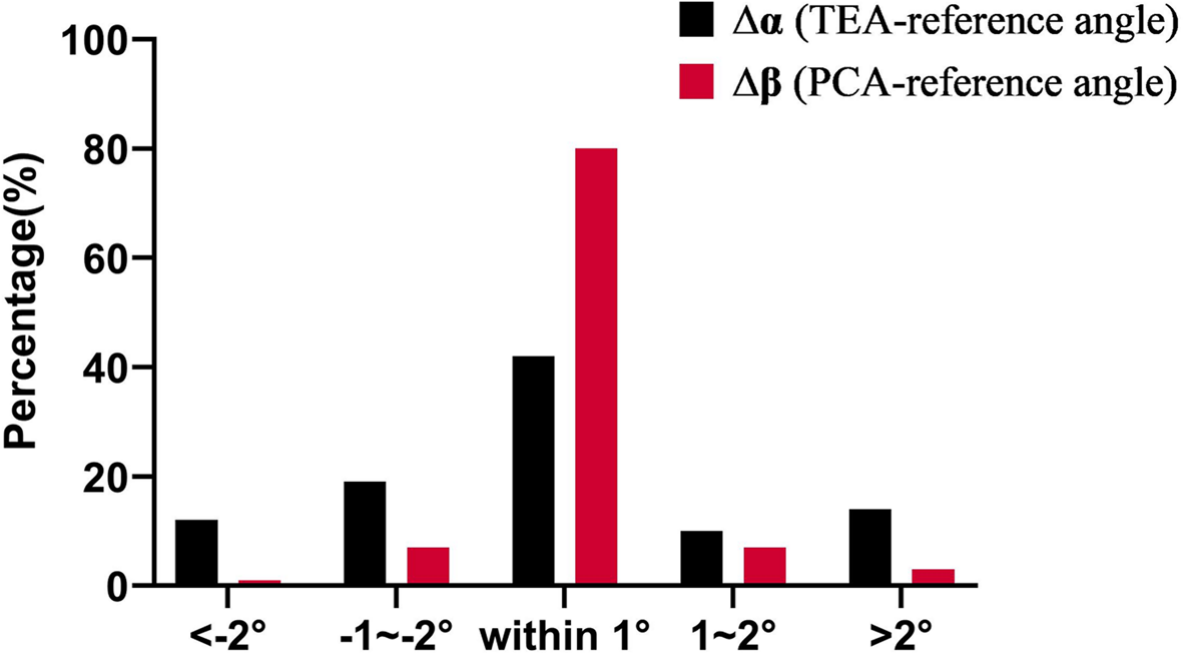
Distribution of the ∆α and ∆β values. The α value is the TEA-reference angle (the angle between the transepicondylar axis and the femoral component axis) when the PCA-reference angle (the angle between the posterior condylar axis and the femoral component axis) is adjusted to 0°; the β value is the PCA-reference angle when the TEA-reference angle is adjusted to 0°. The measured values were α_1_β_1_ for Surgeon-1 and α_2_β_2_ for Surgeon-2. We defined ∆α (black column) as α_1_-α_2_ and ∆β (red column) as β_1_-β_2_

The ICC(2,1) revealed that both the TEA landmark location (α) and the PCA landmark location (β) were consistent across different surgeons (*P* < 0.05). However, the ICC of α was 0.761, and that of β was 0.943 (Table 2). With respect to the inter-differences between surgeons in terms of the TEA-reference angle (∆α), 15 of 56 cases (26.8%, 95% CI: 17.0%–39.6%) had a difference > 2°, and 24 cases (42.9%, 95% CI: 30.5%–56.0%) had a difference within ≤ 1°. In contrast, for the PCA-reference angle (∆β), only 3 of 56 cases (5.4%, 95% CI: 1.8%–14.8%) showed a difference > 2°, whereas 45 cases (80.4%, 95% CI: 68.1%–88.9%) were within ≤ 1° (Fig. 5), indicating that the posterior condylar axis seems to have better consistency.

**Table 2:** Inter-surgeon reliability

*n* = 56	Surgeon-1	Surgeon-2	ICC (2,1) [95%CI]	SEM	MDC	*P*-value
α value	2.28 ± 3.06°	2.36 ± 3.40°	0.761 [0.592–0.860]	2.01°	5.57°	< 0.001
β value	2.19 ± 2.82°	2.22 ± 3.29°	0.943 [0.902–0.966]	1.01°	2.80°	< 0.001

α value, the angle between the transepicondylar axis and the femoral component axis when the angle between the posterior condylar axis and the femoral component axis was adjusted to 0°. β value, the angle between the posterior condylar axis and the femoral component axis when the angle between the transepicondylar axis and the femoral component axis was adjusted to 0°. The values of α and β were defined as positive for external rotation and negative for internal rotation. Quantitative data are presented as means ± SDs. ICC(2,1), two-way random-effects model, absolute agreement, single-measure intraclass correlation coefficient; CI, confidence intervals. SEM, the standard error of measurement; MDC, the minimal detectable change

**Fig. 6:**
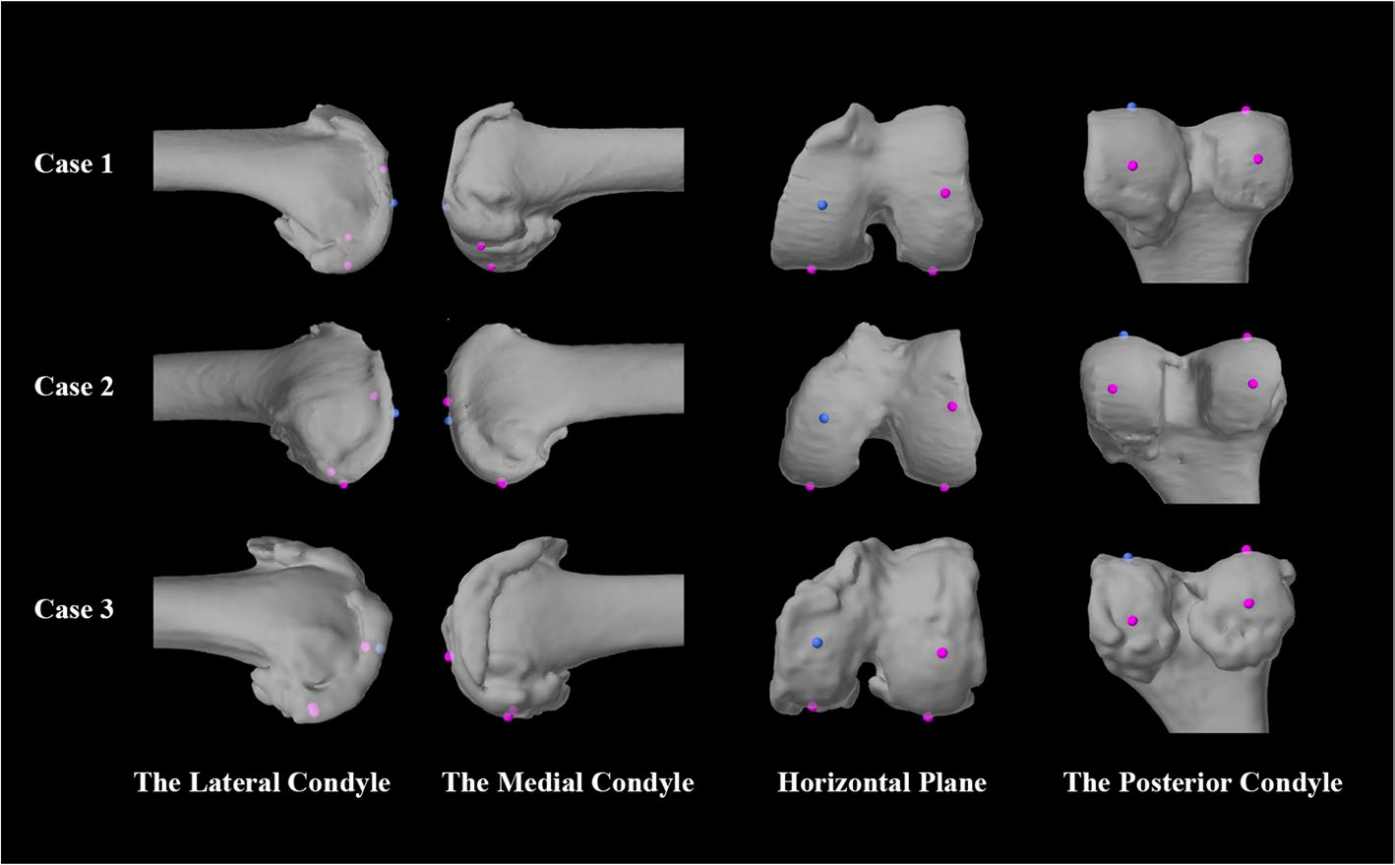
Typical cases with anatomical abnormalities leading to large inter-surgeon differences. This is a screenshot from the MAKO TKA system of three patients’ distal femur models with anatomical abnormalities: Case 1 shows the flat lateral condylar prominence, Case 2 shows two prominences in the medial condyle, and Case 3 shows the hypertrophic posterior condyles. The dots represent anatomical points (the distal femoral point and the medial and lateral posterior condylar points, respectively)

We analyzed cases with large inter-surgeon differences (|∆α| or |∆β|> 2°) and identified anatomical variations contributing to this variability. Three representative cases are shown in Fig. 6.

## Discussion

This is the first study to quantitatively evaluate the differences in femoral component rotation due to landmarks identified by different surgeons directly on the MAKO TKA system. Our data revealed that the difference between the two landmarks’ identification in robotic-assisted TKA planning, which may have caused problems with surgical planning.

Our data revealed that the PCA was significantly more consistently identified than the TEA was (the ICCs of α and β were 0.761 and 0.943, respectively). Jerosch [17] reported that the range of anatomical points chosen by each surgeon during the operation varied by 22.3 mm on the medial epicondyle and 13.8 mm on the lateral epicondyle; even experienced surgeons experience difficulty in identifying its location [18], which was in line with our results. Moreover, our results indicated that, in 26.8% of patients, the observer difference was > 2° (up to 12°) for the TEA-reference angle, which may significantly affect the femoral component rotation. Numerous studies have shown that incorrect rotation of the femoral component is associated with many complications, including patellofemoral maltracking, anterior knee pain, stiffness, flexion instability, and abnormal torsional stress on the tibial component, leading to wear or loosening, and cam post impingement in a posterior-stabilized knee [19–24]. Therefore, the difference caused by landmark selection warrants attention, considering the significant observer difference for the TEA-reference angle. Although the TEA is considered the most accurate landmark for locating the flexion axis of the knee [9], such a large variation can confuse doctors during surgery.

On the other hand, on the basis of our data, the observer difference was within 1° for the PCA-reference angle in 80.4% of patients; thus, the PCA seems to be a more informative landmark under robotic-assisted TKA. In general, the PCA with 3° internal rotation is usually used to determine the external rotation of the femoral component during surgery [9]. However, ethnicity also affects the angle between the PCA and TEA [25]; thus, 3° is not accurate for all patients. In addition, in the context of robotic-assisted TKA, the ideal PCA-reference angle has not been established. Therefore, the experience of femoral rotation in manual TKA may not apply to robotic-assisted TKA, and we recommend the use of double-landmark cross-checking.

Surgeons who choose the TEA may believe that the TEA is closer to the flexion axis of the knee [8], whereas the PCA is based on 3–4° internally rotated in relation to the TEA [9]. However, this anatomical-based definition does not seem important in the robotic ligament balancing workflow, and we may need a more stable metric to evaluate the parameter boundaries for each surgery. It seems that the PCA is more reliable with respect to this criterion. However, this does not mean that the TEA should be out of history, but rather that when the TEA is chosen as a reference, more attention should be given to the accuracy of landmark identification, especially when it is too far out of line compared with experience.

We analyzed cases with excessive differences in landmark location and selected three typical samples. Three representative anatomically abnormal types of the medial and lateral condyles were included for the following typical patients: first, the lateral condylar prominence was so flat that the most prominent point was not recognizable (Case 1); second, two prominences were present in the medial condylar, leading to uncertainty in selecting the correct sulcus (Case 2); and third, the hypertrophic posterior condyles exhibited irregular morphology, complicating the identification of the definitive most posterior point on the medial side (Case 3). In such cases with ambiguous anatomy, surgeons choosing different anatomical points resulted in angular differences exceeding 2°. Thus, surgeons should focus more intently on landmark location during the second preoperative examination.

There are several limitations in our study. First, only two surgeons were compared, and the differences may vary from surgeon to surgeon; however, the tendency for the TEA-reference angle to have a greater dispersion is clear. Second, only the MAKO TKA system was used, and the results may vary among other robots. Finally, the sample size was small; thus, large-sample studies are needed in the future.

## Conclusion

The inter-surgeon consistency of identification of the PCA was significantly greater than that of the TEA in the context of robotic-assisted TKA planning. To mitigate the risk of inappropriate femoral component rotation, surgeons should verify landmark positions, particularly in patients with anatomical abnormalities of the distal femur. Utilizing the more consistent posterior condylar axis as a primary reference or employing a dual-landmark cross-checking strategy within the robotic system is recommended to enhance the reliability of rotational alignment.

## Data Availability

The datasets used and/or analyzed during the current study are available from the corresponding author upon reasonable request.

## Abbreviations

TKA: Total knee arthroplasty
TEA: Transepicondylar axis
PCA: Posterior condylar axis
TEA-reference angle: The angle between the TEA and the femoral component axis
PCA-reference angle: The angle between the PCA and the femoral component axis
ICC: Inter-surgeon intraclass correlation coefficient
